# The occurrence of helminth parasites in the gastrointestinal of catfish (*Silurus glanis Linnaeus* 1758) from the Zarrine-roud river, Iran

**Published:** 2012

**Authors:** Mohammad Yakhchali, Ali-Asghar Tehrani, Mozafar Ghoreishi

**Affiliations:** 1 *Department of Pathobiology, Parasitology division, Faculty of Veterinary Medicine, Urmia University, Urmia, Iran; *; 2 *Department of Pathobiology, Pathology division, Faculty of Veterinary Medicine, Urmia University, Urmia, Iran;*; 3 *General Veterinary practitioner in Bookan, Iran.*

**Keywords:** Helminths, *Silurus glanis*, Zarrine-roud, Iran

## Abstract

This investigation was undertaken to verify the prevalence of helminths parasites in the gastrointestinal tract of catfish. A total number of 116 catfish (*Silurus glanis*) were collected from Zarrine-roud river and examined for helminths. Fish were examined after washing contents of gastrointestinal tract and observed for the presence of helminths using a stereo microscope and a light microscope. Results indicated that 18.96% of the examined catfish were infected with digenean trematodes including *Orientocreadium siluri* (27%), *Crowcrocoecum skrjabini* (39%), and cestode *Bothriocephalus gowkongensis *(34%). All the parasites were found in the intestine. Mid-gut followed by foregut appeared to be the most commonly infected parts of the alimentary tract of hosts. The results showed that there was a significant correlation between infection rate, catfish body size, and weight (*P* < 0.05).

## Introduction

Determination of the rate of parasitic infections in fish especially gastrointestinal helminths infection plays an important role in epidemiology of fish parasitic diseases and in particular their routes of transmission to other hosts. Helminths parasites frequently occur within the viscera and body cavity of catfish especially intestine, therefore, they usually damage the gastrointestinal tract. 

The first catfish to be scientifically recorded (synonymous to ScotCat) is the *Silurus glanis* (Linnaeus 1758). *Silurus glanis* was originally found in the lakes of Europe and Sweden, and also there are two reports from Iran.^1^ This species of catfish exists in different districts of Iran including Sefid-roud and Anzali wetland around Caspian sea, Zarrine-roud, Simine-roud river, Aras dam in West Azerbaijan^2^ and Khuzestan marshes. ^2^^-^^4^ The Zarrine-roud river is located in southeast of Urmia lake in northwest of Iran (35°58´-39°46´N, 44°33´E) and its length is about 302 km.

Catfish has commercial value in this region and its ecological role as a predator of other fish in the ecosystem is well known. The present study was undertaken to identify species diversity of gastrointestinal helminths of catfish and evaluate their prevalence in the Zarrine-roud river.

## Materials and Methods

Over a one-year period, 116 specimens of catfish (*S. glanis*) were collected from the Zarrine-roud river and examined for common helminths. Upon collection, the fish were placed in seine net bag and washed within the flowing water to clean from mud. Age, weight and body length of fish as well as the water temperature were recorded during each sampling (Table 1).

**Table 1:** The infection percentage of the examined catfish with helminths based on their age (year), weight (kg) and body length (cm).

**Number of catfish**	**Infected catfish (%)**	**Age (year)**	**Weight (kg)**	**Body length** ** (cm)**
12	9 (7.76)	<1	0.5-1	30-50
70	0 (0)	1-4	2-3	50-70
27	13 (11.20)	4-9	3-7	70-140
7	0 (0)	9-10	7-9	140-300
Total (116)	22 (18.96)	-	-	-

Fish were examined after washing contents off gastrointestinal tract and observed for the presence of helminths under a stereo microscope and a light microscope (125×).^5^ The key identification of Bykhovskaya-Pavloskay *et al.* was used to classify the observed helminths.^6^

Statistical evaluation of data was performed using *t*-test with confidence interval at 95% in SPSS statistical package for Windows (Version 11.5, SPSS Inc., Chicago, USA).

## Results

**Fig. 1:**
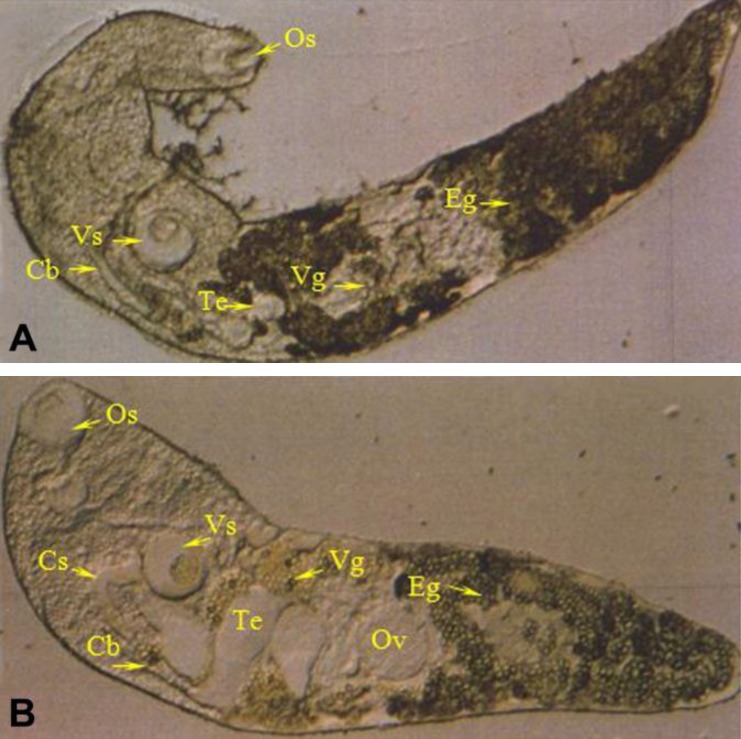
Helminth removed from the intestine of naturally infected catfish. **A**, *Crowcrocoecum skrjabini ***B**:* Orientocreadium siluri* (125×). (Cb: caeca branch, Cs: cirrus sac, Eg: egg, Os: oral sucker, Ov: ovary, Te: testis, Vg: vitelline glands, Vs: ventral sucker).

Overall prevalence of helminths infection was 18.96%. Infected catfish were 30-50 (min.) cm and 70-140 (max.) cm in length, weighting 0.5 –1 (min.) kg and 3-7 (max.) kg, age ranging from less than 1 and 4-9 year-old. There was a significant correlation between infection rate and the catfish body size and weight (*P* < 0.05). Three species of helminths were identified as *Orientocreadium siluri* (27%),* Crowcrocoecum skrjabini* (39%), and* Bothriocephalus gowkongensis* (34%) (Table 2) (Fig. 1). The most infected parts of gastrointestinal tract were foregut and midgut. The highest prevalence was 11.20% with water temperature of 7-10 °C between November to December. 

**Table 2:** Key identification of recovered helminths from naturally infected catfish in Zarrine-roud river.

**Parasite**	**Mean size (mm)**		**Identified features**
*Bothriocephalus gowkongensis*	487.1-818.5	Scolex: Bothria narrow and expanded at end, Testis: 54-60, Ovary: compact, Uterine sac spherical.	
*Crowcrocoecum skrjabini*	0.9-1.6 × 0.27-1.05	Tegument: Thick and smooth, Sucker: Ventral larger than oral sucker, Intestine: Caeca with two branches, Testis: In space between caeca, Vitelline glands: lying in second half of body.	
*Orientocreadium siluri*	1.1-2.1 × 0.13-0.19	Tegument: densely spinose in front half, Suckers: Ventral sucker relatively small, Intestine: Caeca with two branches, Vitelline glands: extended from posterior margin of ventral sucker.	

## Discussion

Upon dissection of fish, helminths from the gut are often the most obvious parasites to be seen. They may occur in large numbers and several species of digeneans, cestodes, nematodes, and acanthocephalans may be present in one individual host. Despite of this, helminths are rarely implicated as causes of disease. Of those, cestodes are the most important parasites.^7^ The parasitic helminths fauna and pathology of siluridae have rarely been reported. Early studies have reported on the presence of specific group and disease outbreaks in aquarium fish.^8^

In the present study, the highest mean prevalence of trematodes was in close agreement with the results of Mortezaei *et al.* and Veljovic* et al.*^2^^,^^10^ Dezfuli *et al.* and Oyamada *et al.* noted that approximately the same 50% of the catfish were infected, all of which were 30 cm or longer in body length.^11^^,^^12^ Hoffnagle *et al*. has reported three trematodes and three cestodes (including* Proteocephalus fragile*) in catfish (*Ictalurus furcatus*).^13^ Additionally, in this investigation mean abundance of identified parasites was relatively high between November and December. This finding thus lends the data obtained by Amin.^14^

In this study, the majority of lesions noted in catfish were unlikely to have an impact on host survival. However, several of the recorded species can be pathogenic and cause economic loss under certain conditions. Pathogenicity due to multiple infections was minimal, apparently limited to denudation of the mucosal folds.

This investigation, as well as previous investigations on catfish, demonstrated that the infected intestine enlarged and dilated at the point of attachment, appeared as a white dot. Yakhchali and Tehrani noted that histopathological findings revealed a severe infiltration of the leukocytes, especially, lymphocytes and eosinophil granular cells (ECGs) in the mucosa membrane.^15^ Scolex was surrounded by degeneration and necrosis of enterocytes, and connective tissue which was originated from the submucosa. Although it appeared that the adult digeneans and cestodes found in catfish were parasites of the alimentary tract, in a few cases were implicated as primary cause of disease. Because of helminths attachment to the host intestine, they do not elicit a severe host reaction. Furthermore, wild catfish are well adapted to parasites invasion, and lesions are generally restricted to mild inflammatory change or hemorrhages at sites of attachment or feeding.^7^
